# Ratcheting by Stochastic Resetting With Fat‐Tailed Time Distributions

**DOI:** 10.1002/cphc.202400313

**Published:** 2024-10-22

**Authors:** Jianli Liu, Yunyun Li, Pulak K. Ghosh, Shubhadip Nayak, Fabio Marchesoni

**Affiliations:** ^1^ IMOE Key Laboratory of Advanced Mico-Structured Materials and Shanghai Key Laboratory of Special Artificial Microstructure Materials and Technology School of Physics Science and Engineering Tongji University Shanghai 200092 China; ^2^ Department of Chemistry Presidency University Kolkata 700073 India; ^3^ Dipartimento di Fisica Università di Camerino, I- 62032 Camerino Italy

**Keywords:** Stochastic resetting, Brownian motors, Superdiffusion, Lévy flights

## Abstract

We investigated both numerically and analytically the drift of a Brownian particle in a ratchet potential under stochastic resetting with fat‐tailed distributions. As a study case we chose a Pareto time distribution with tail index *β*. We observed that for 1/2<β<1 rectification occurs even if for β<1 the mean resetting time is infinite. However, for β≤1/2 rectification is completely suppressed. For low noise levels, the drift speed attains a maximum for *β* immediately above 1, that is for finite but large mean resetting times. In correspondence with such an optimal drift the particle diffusion over the ratchet potential turns from normal to superdiffusive, a property also related to the fat tails of the resetting time distribution.

## Graphical abstract

A diffusing swimmer can autonomously rectify its random motion in the absence of external time‐dependent fields of force or gradients, simply by switching on and off its internal engine to adjust to the spatial asymmetry of the substrate. For appropriate power‐law distributions of its switching times the swimmer drifts superdiffusively with exponent close to two.

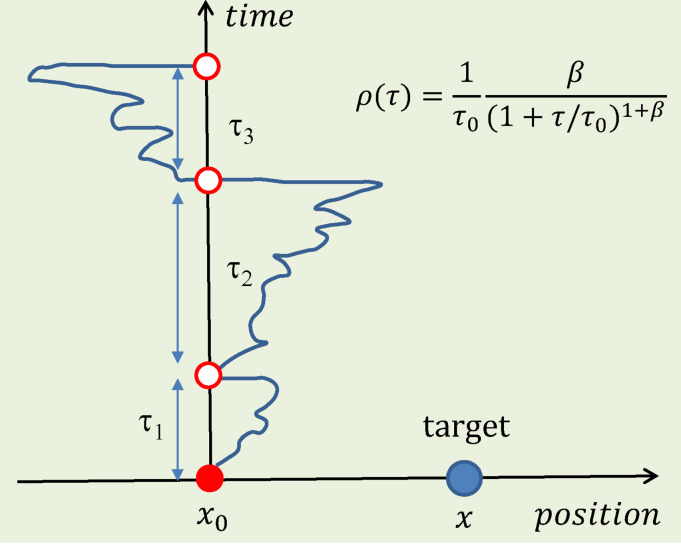

## 1 Introduction

The term stochastic resetting (SR) refers to the sudden interruption of a stochastic process after random time intervals, *τ*, followed by its starting anew (possibly after a finite latency time), under same dynamical conditions.^[1]^ This class of non‐equilibrium stationary processes found applications in searching contexts,^[2]^ optimization of randomized computer algorithms,^[3]^ and in many biophysical problems.[^4^, ^5^] The notion of SR owes its initial popularity to the observation that under SR the otherwise infinite mean first passage time (MFPT) for a one‐dimensional (1D) free Brownian particle^[6]^ to diffuse from an injection to an assigned target point becomes finite. Most notably, it has been reported that such MFPT can be minimized for an optimal choice of the mean resetting time, ⟨τ⟩. [^7^, ^8^] Standard stochastic methods[^6^, ^9^] can be generalized to study diffusion under SR, for instance, to calculate the MFPT of a reset particle out of a one‐dimensional (1D) domain,^[10]^ potential well,^[11]^ or cavity.^[12]^ In general, SR speeds up (slows down) diffusive processes characterized by random characteristic times with standard deviation larger (smaller) than the respective averages.^[5]^

**Figure 1:**
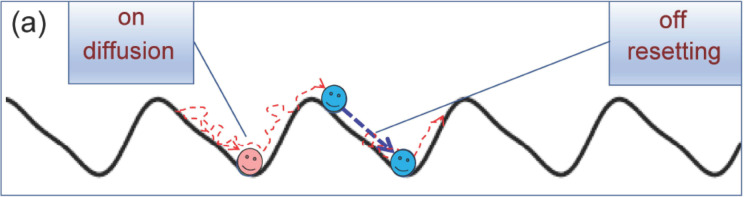
Autonomous ratcheting by stochastic resetting: schematics.

A variation of this mechanism is *autonomous* SR,^[13]^ whereby a small motile tracer (like a bacterium or a micro‐robot^[14]^) of coordinate *x* undergoes overdamped Brownian motion on a 1D substrate by switching its internal engine on and off. Let the substrate be represented by a 1D periodic potential, *V*(*x*), of period *x_L_*, and assume for simplicity that the potential unit cells have one minimum each at $x_{n}=x_{0}+nx_{L}$ , with n=0,±1,⋯ . At resetting, the particle stops diffusing and falls instantaneously at the bottom of the potential well it was in and it resumes diffusing from there after an arbitrary small latency time (Figure 1). If we further assume that the barriers separating two adjacent potential minima are asymmetric under mirror reflection, i. e., $V\left(x-x_{0}\right)\neq V\left(-x+x_{0}\right)$ (ratchet potential[^15^, ^16^, ^17^, ^18^, ^19^]), then (i) SR suffices to *rectify* the particle's diffusion. The net drift reaches a maximum for an optimal value of ⟨τ⟩ ; (ii) SR suppresses the particle's spatial diffusion [quantified by its mean square displacement (MSD)], which however retains its *normal* character (i. e., its asymptotic linear dependence on time). The relevant diffusion constant increases sharply with ⟨τ⟩ in correspondence with the maximum of the drift speed.^[13]^ As a consequence, the diffusing tracer can autonomously rectify its random motion in the absence of external time‐dependent fields of force or gradients, simply by time‐operating its internal engine to adjust to the spatial asymmetry of the substrate.

In both SR applications, namely either to control the MFPT of an unbounded Brownian particle diffusing between two given points or to rectify its motion along an asymmetric substrate, the control parameter of the resetting protocol is generally chosen to be the mean SR time, ⟨τ⟩ , independently of the actual *τ* distribution.^[8]^ Clearly, this approach is no longer tenable in the case of a fat‐tailed *τ*‐distribution, when ⟨τ⟩ may diverge. By adopting a Pareto's power‐law distribution for the SR times, we investigated, both analytically and numerically, under what conditions SR can be a useful stochastic control tool also for infinite ⟨τ⟩ .

The contents of this paper is organized as follows. In Sec. 2 we formulate a basic Brownian diffusion model and the SR time distribution adopted in our numerical simulations, namely a Pareto (Type I) distribution. In Sec. 3 we focus on some peculiar properties of the SR statistics due to the fat tails of the chosen *τ*‐distribution, with and without substrate potential. In Sec. 4 we analyze the dependence of the drift speed on the Pareto *τ*‐distribution parameters. We conclude that SR rectification may occur also for infinite ⟨τ⟩ . In Sec. 5 we investigate the particle diffusivity under SR and observe that for large but finite values of ⟨τ⟩ the particle's dynamics may become superdiffusive, a phenomenon never reported for exponential *τ*‐distributions. Finally, in Sec. 6 we draw some concluding remarks about possible generalizations and applications of the present model.

## 2 Model

**Figure 2:**
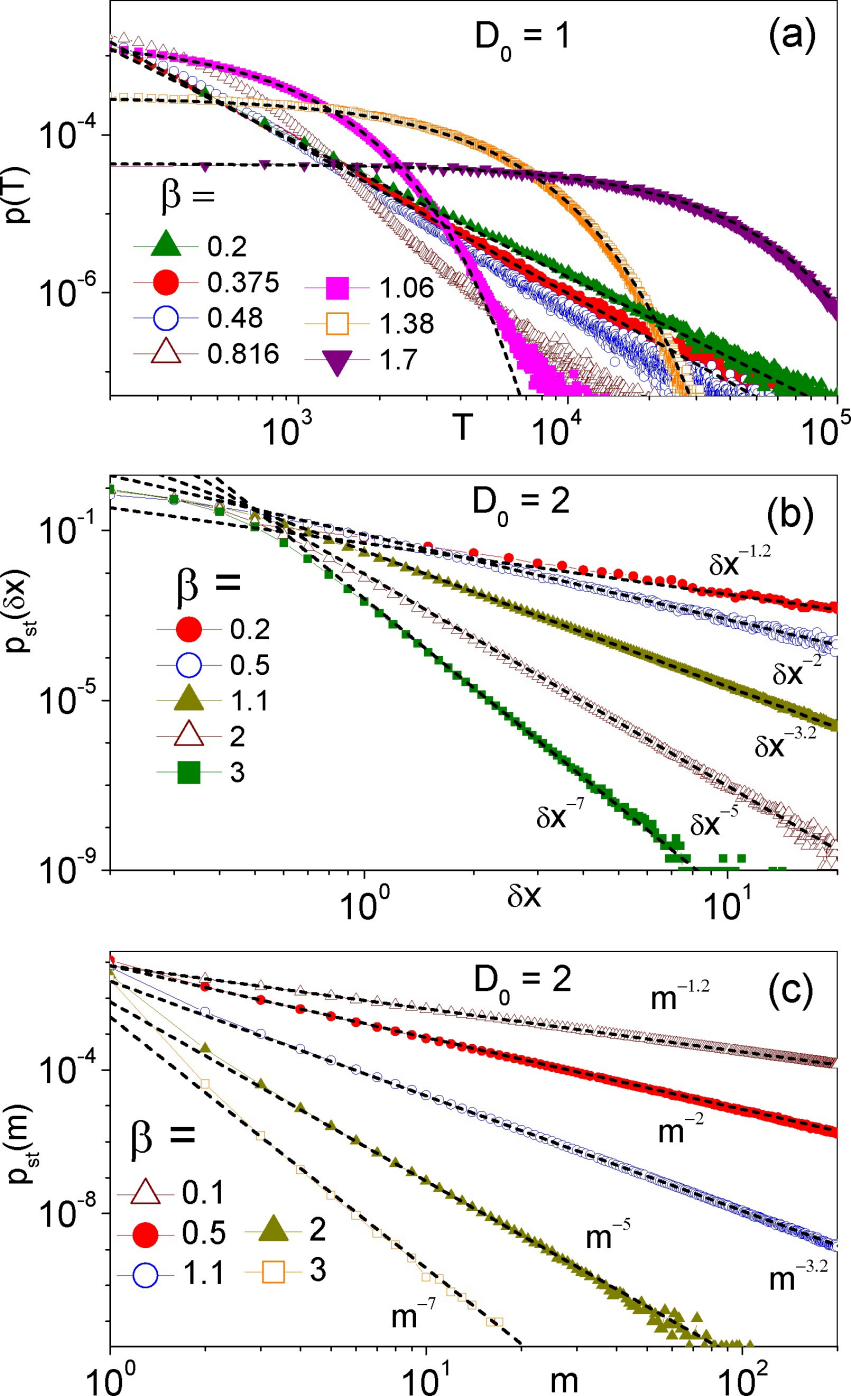
Pareto resetting statistics: (a) distribution of the FPT's, *T*, for a free Brownian particle with *D*_0_ *=*1 to diffuse a distance L=10 under Pareto SR. The resetting distance distributions with and without ratchet potential are plotted respectively in (b) and (c) for *D*_0_ *=*2. In (b) *δx* is the particle's net displacement between two consecutive resettings; in (c) *m* denotes the distance in units of *x_L_* between two consecutive resetting potential minima. Dashes lines represent the fitting power laws with exponents $-\left(3/2+\beta \right)$ in (a) and $-\left(1+2\beta \right)$ in (b) and (c); data sets for β>1 in (a) have been fitted by exponential curves. In all simulations the SR time was distributed according to Eq. (3) with $\tau_{0}=0.01$ ; the ratchet potential, when present, is *V*(*x*) of Eq. (2) with $x_{L}=1$ .

**Figure 3:**
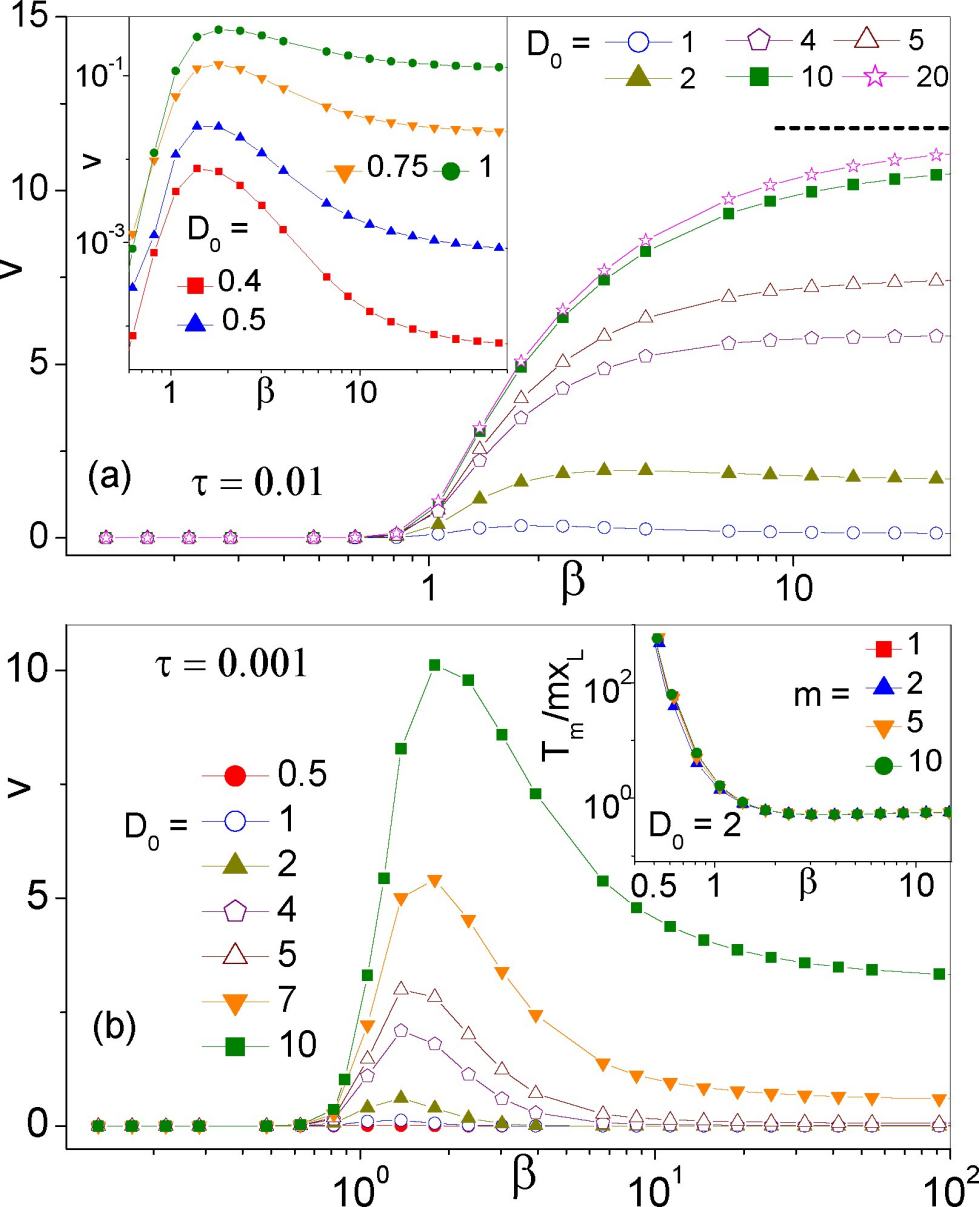
Drift speed, *v* vs. *β*, in the ratchet potential *V*(*x*) of Eq. (2) with Pareto *τ* statistics, Eq. (3), for $x_{L}=1$ , $\tau_{0}=0.01$ (a) and 0.001 (b), and different *D*_0_ (see legends). In the inset of (b) we report the reciprocal of *v* (see text) vs. *β* to better illustrate rectification suppression for β<1/2 . The horizontal dashed line in (a) represents the estimate $v=0.12x_{L}/\tau_{0}$ valid for large *β* and *D*_0_ (see Sec. 4).

**Figure 4:**
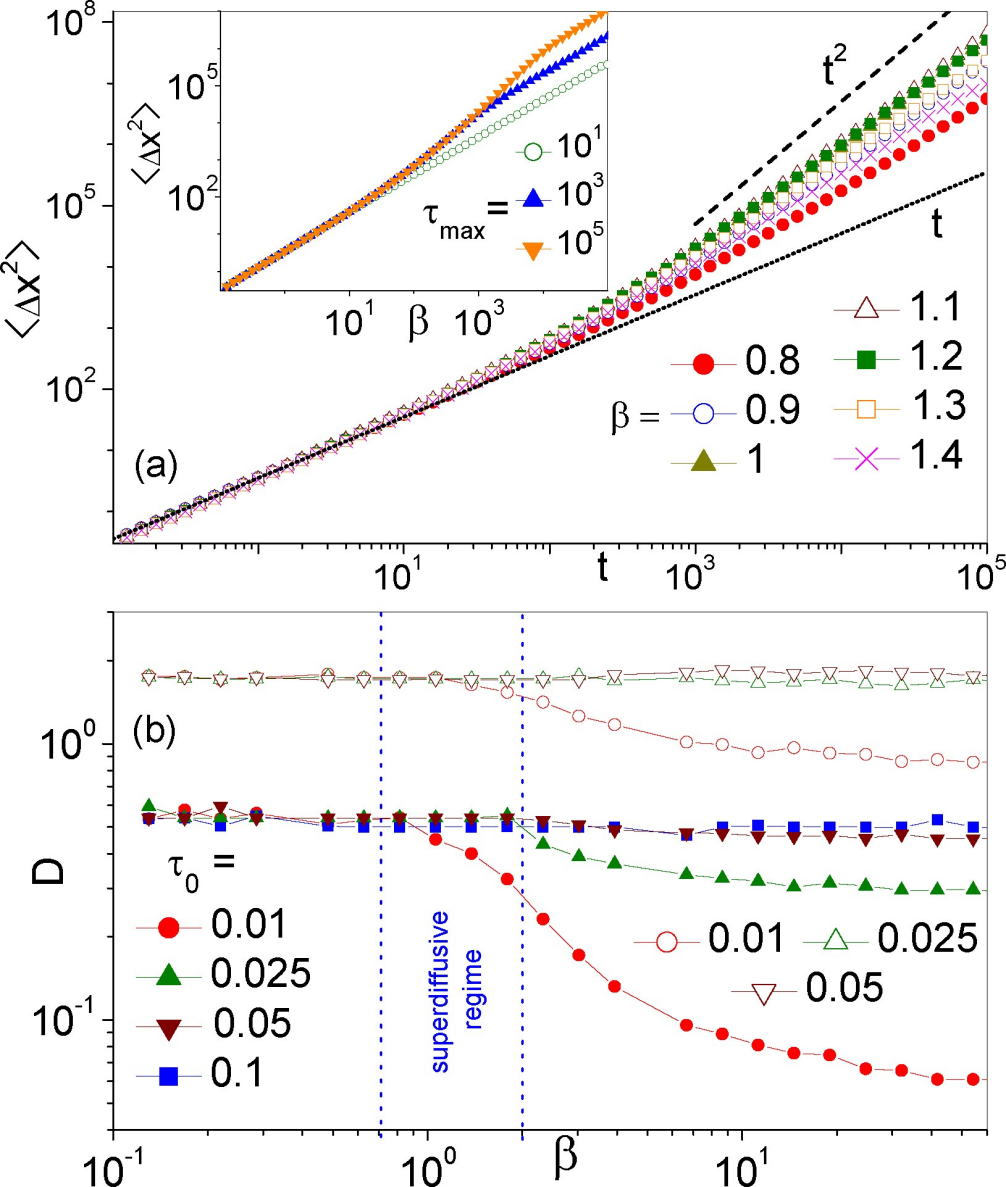
Diffusion of a particle in the ratchet potential *V*(*x*) of Eq. (2) with Pareto SR time statistics, Eq. (3). (a) $\left\langle \Delta x^{2}\right\rangle$ , Eq. (4), vs. *t* for *D*_0_ *=*2, $\tau_{0}=0.01$ , and different *β* (see legends). In the inset, the curve $\left\langle \Delta x^{2}\right\rangle$ vs. *t* is plotted for β=1.1 and Pareto *τ*‐distributions truncated at $\tau =\tau_{\max}$ (see text). (b) Diffusion constant, *D*, of Eq. (4) vs. *β*, for *D*_0_ *=*1 (empty symbols) and 2 (solid symbols), and different *τ*_0_ (see legend). In the superdiffusive regime (marked *β* range), *D* was extracted from the linear branch of $\left\langle \Delta x^{2}\right\rangle$ curves [see (a)]. In both panels $x_{L}=1$ .

The simulated particle dynamics was formulated in terms of the overdamped Langevin equation (LE),
(1)

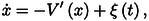




where $\xi \left(t\right)$ denotes a stationary zero‐mean valued Gaussian noise with autocorrelation $\left\langle \xi \left(t\right)\xi \left(0\right)\right\rangle =2D_{0}\delta \left(t\right)$ (white noise) and *V*(*x*) is the standard ratchet potential,[^15^, ^16^]

(2) $$V\left(x\right)=\sin\left(2\pi x/L\right)+\left(1/4\right)\sin\left(4\pi x/L\right),$$

with asymmetric barriers of height $\Delta V=\left(3/2\right){\left(1+2/\sqrt{3}\right)}^{1/2}≃2.20$ . The potential unit cell [$0,x_{L}]$ has a maximum (barrier) at $x_{b}=\left(x_{L}/2\pi \right)$ arccos $\left[\left(\sqrt{3}-1\right)/2\right]≃0.19x_{L}$ and a minimum (well bottom) at $x_{0}=x_{L}-x_{b}≃0.81x_{L}$ , with curvatures 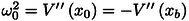 $={\left(2\pi /x_{L}\right)}^{2}{\left(3\sqrt{3}/2\right)}^{1/2}≃63.6/x_{L}^{2}$ , see Figure 1. The asymmetric potential wells have right/left slopes of different lengths, $x_{\left(R,L\right)}$ , with $x_{\left(L\right)}=x_{0}-x_{b}=x_{L}-x_{\left(R\right)}≃0.62x_{L}$ . In addition to the thermal fluctuations and the ratchet potential, the particle is subjected to instantaneous resetting (with zero latency time) at the local minimum after a random time, *τ*, with Pareto (Type I) probability density function (pdf).

(3) $$\rho \left(\tau \right)=\left\{\begin{array}{cc} \frac{\beta }{\tau_{0}}{\left(\frac{\tau_{0}}{\tau }\right)}^{1+\beta }\; & \mathrm{for}\;\tau \geq \tau_{0}\;\\ 0\; & \mathrm{for}\;\tau <\tau_{0},\; \end{array}\right.$$

of positive scale, *τ*_0_, and shape parameter (or tail index) *β*. The main conclusions of the present work can be easily extended to a Lomax distribution with same scale and shape parameters or even to other fat‐tailed *τ* distributions. Along with the restart protocol, the Eq. (1) was numerically integrated by means of a standard Milstein scheme,^[20]^ to compute the FPT's, $T_{\delta x}$ (*T_m_*), for a particle to diffuse a distance *δx* (*mx_L_*) from the resetting point (Figure 2), the drift speed $v=\lim_{t\to \infty }\left[\left\langle x\left(t\right)\right\rangle -x\left(0\right)\right]/t$ (Figure 3), and the asymptotic MSD,

(4) $$\left\langle \Delta x^{2}\left(t\right)\right\rangle =\left\langle x^{2}\left(t\right)\right\rangle -v^{2}t^{2}\equiv 2Dt,$$

of a particle under stationary conditions (with or without SR) (Figure 4). Particular attention was paid to choose appropriate integration steps and running times, typically *δ* $t=10^{-3}$ and $t=10^{6}$ . However, more accurate estimates of the above dynamical quantifiers required much longer simulation runs and shorter integration steps, respectively for small values of *β* and *τ*_0_.

## 3 Stochastic Resetting Statistics

In the current SR literature the *τ*‐distribution is often assumed to be $\rho \left(\tau \right)=\exp\left(-\tau /\left\langle \tau \right\rangle \right)/\left\langle \tau \right\rangle$ (constant restart rate, ${\left\langle \tau \right\rangle }^{-1}$ ), or $\rho \left(\tau \right)=\delta \left(\tau -\left\langle \tau \right\rangle \right)$ (sharp restart). In both cases, the MFPT for an unbounded Brownian particle to diffuse a distance *L*, say, $x_{0}\to x_{0}+L$ , is “regularized”,^[7]^ i. e.,

(5) $$\left\langle T\right\rangle =\left\langle \tau \right\rangle \exp\left[\left(L/\sqrt{D_{0}\left\langle \tau \right\rangle }\right)-1\right].$$

Other exponential distributions, $\rho \left(\tau \right)$ , yield similar predictions as long as $\left\langle \tau \right\rangle$ is finite; hence the choice of $\left\langle \tau \right\rangle$ as a natural SR control parameter.^[10]^ By the same token, the particle displacement, *δ* $x=x-x_{0}$ , assumes a stationary distribution.^[7]^ For a constant SR rate, $\left\langle \tau \right\rangle$ , such a distribution reads

(6) $$p_{st}\left(\delta x\right)=\int_{0}^{\infty }p\left(\delta x;\tau \right)\rho \left(\tau \right)d\tau =\frac{1}{\sqrt{4D_{0}\left\langle \tau \right\rangle }}e^{-\frac{\left|\delta x\right|}{\sqrt{D_{0}\left\langle \tau \right\rangle }}},$$

where $p\left(\delta x;t\right)={\left(4\pi D_{0}t\right)}^{-1/2}\exp[-\left({\delta x)}^{2}/4D_{0}t\right]$ is the non‐stationary Gaussian pdf of the same particle at time *t* in the absence of SR. Relevant to our presentation is also recalling that the distribution of the FPT for the process $x_{0}\to x_{0}+L$ , *p*(*T*), can also be computed analytically.^[7]^ In the limit of interest here, $L\gg \sqrt{D_{0}\left\langle \tau \right\rangle }$ , *p*(*T*) decays exponentially, that is

(7) $$p\left(T\right)∼e^{-T/\left\langle T\right\rangle }/\left\langle T\right\rangle .$$

For the Pareto *τ*‐distribution of Eq. (3), we notice that

(8) $$\left\langle \tau \right\rangle =\frac{\beta }{\beta -1}\tau_{0},$$

that is, $\left\langle \tau \right\rangle$ is infinite for β≤1 . The question is then to determine the SR effects on the Brownian dynamics of Eq. (1) over the entire range of the tail index, *β*. To this purpose we first considered the predictions of Eqs. (6) and (7) for the diffusion of a free Brownian particle with $V\left(x\right)\equiv 0$ . Replacing the Pareto distribution, Eq. (3), into Eq. (6) yields, in the limit $x\gg \sqrt{D_{0}\tau_{0}}$ ,

(9) $$p_{\mathrm{st}}\left(\delta x\right)=\frac{\beta }{4\pi D_{0}}{\left(\frac{\sqrt{4D_{0}\tau_{0}}}{\delta x}\right)}^{1+2\beta }.$$

The distribution of a free particle position subject to SR with Pareto time statistics is thus stationary, but its mean, $\left\langle x\right\rangle$ , is infinite for β<1/2 . The above power‐law decay of $p_{\mathrm{st}}\left(\delta x\right)$ is confirmed by the simulation data displayed in Figure 2(b). In passing, we anticipate that a decay law with the same exponent was obtained also for particles diffusing on the ratchet substrate of Eq. (2). In that case the particle displacement was measured as the distance between two consecutive restarting *V*(*x*) minima, $x_{0}\to x_{0}+mx_{L}$ , namely *δ* $x=mx_{L}$ . In Figure 2(c) the resulting jump distributions $p_{\mathrm{st}}\left(m\right)$ are plotted for m≥0 , only, because the positive and negative power‐law tails of $p_{\mathrm{st}}\left(m\right)$ appear to coincide, that is, for large displacements, $p_{\mathrm{st}}\left(-m\right)=p_{\mathrm{st}}\left(m\right)$ .

Finally, coming back to the diffusion of a free particle with $V\left(x\right)\equiv 0$ , we numerically computed the FPT distributions with the Pareto *τ*‐distribution of Eq. (3). Our numerical results are displayed in Figure 2(a). One observes immediately that for β>1 , *p*(*T*) decays exponentially, as predicted in Eq. (7), whereas for β<1/2 it decays according to the power law $1/T^{3/2+\beta }$ . On increasing *β* from 1/2 to 1, an exponential decay for small *T* coexists with a residual power‐law tail, the former gradually taking over the latter.

The exponential decay of *p*(*T*) is consistent with the theoretical prediction of Eq. (7) as for β>1 the mean SR time, $\left\langle \tau \right\rangle$ , of Eq. (8) is finite. Vice versa, as $\left\langle \tau \right\rangle$ diverges, *p*(*T*) may be taken approximately proportional to $f\left(T\right)\mathrm{Prob}\left(T<\tau \right)$ , where $f\left(T\right)={\left(L^{2}/4\pi D_{0}\right)}^{1/2}\exp\left(-L^{2}/4D_{0}T\right)/T^{3/2}$ is the Lévy‐Smirnov distribution of the particle's FPT in the absence of resetting,^[6]^ and $\mathrm{Prob}\left(T<\tau \right)=\int_{T}^{\infty }\rho \left(\tau \right)d\tau$ denotes the probability that *T* is smaller than the SR time. Stated otherwise, we require that the no‐SR FPT distribution, *f*(*T*), is conditional to the FPT, *T*, being not larger than the actual resetting time, *τ*. In the limit of interest for our presentation, $L\gg \sqrt{D_{0}\tau_{0}}$ , an explicit integration yields

(10) $$p\left(T\right)≃\frac{1}{\tau_{0}}{\left(\frac{L^{2}}{4\pi D_{0}\tau_{0}}\right)}^{\frac{1}{2}}{\left(\frac{\tau_{0}}{T}\right)}^{\frac{3}{2}+\beta }.$$

This estimate for *p*(*T*) explains the power‐law tails of the curves with β<1 shown in Figure 2(a). Moreover, based on it, one expects that $\left\langle T\right\rangle$ diverges for β<1/2 . As a matter of fact, our numerical simulations point to the existence of particle's trajectories that fail to hit the target at *x*_0+*L*_ even after exceedingly long computer running times (i. e., up to 10^11^ time units).

Similarly to the $p_{\mathrm{st}}\left(\delta x\right)$ distributions, the power‐law decay of *p*(*T*) holds good also in the presence of the periodic substrate potential, *V*(*x*), as discussed below in Sec. 4. In conclusion, SR with a Pareto *τ*‐distribution might still provide a useful stochastic control technique also for infinite $\left\langle \tau \right\rangle$ , but only in the tail index range 1/2<β<1 . As a final remark, we notice that for β→∞ , the mean reset time tends to the Pareto scale parameter, $\left\langle \tau \right\rangle \to \tau_{0}$ . In such a limit the formulas of Eqs. (5)‐(7) are a good approximation for our model, too, under the conditions that $x_{L}\gg \sqrt{D_{0}\tau_{0}}$ or $L\gg \sqrt{D_{0}\tau_{0}}$ , as appropriate.

## 4 Rectification by Stochastic Resetting

We address now the effect of rectification by SR in the asymmetric potential *V*(*x*). In Ref. [13] this mechanism was analyzed in detail for exponential and periodic SR time statistics. The most natural descriptor, the drift speed *v* defined in Sec. 2, is plotted versus *β* in Figure 3 for Pareto *τ*‐distributions with small *τ*_0_. The positive sign of *v* is determined by the profile of *V*(*x*) (Figure 1) and is related to the skewness of the jump distributions $p_{\mathrm{st}}\left(m\right)$ [Figure 2(c)], which deviate from a power‐law decay for very small values of *m*, typically m<3 , with $p_{\mathrm{st}}\left(-m\right)<p_{\mathrm{st}}\left(m\right)$ . As anticipated at the end of Sec. 3, for large *β* we recovered the results of Ref. [13] with $\left\langle \tau \right\rangle =\tau_{0}$ . In particular, in the regime of strong noise, $D_{0}\gg \Delta V$ , the drift attains its maximum value $v=\left(x_{\left(L\right)}-x_{\left(R\right)}\right)/2\tau_{0}$ . Most remarkably, *v* drops close to zero for *β* ≲1 , that is as the mean SR time diverges. This behavior comes as no surprise, because rectification in a ratchet potential only occurs in the presence of a time modulation, represented here by the SR protocol, and increasing the modulation time scale only weakens the effect.^[15]^

At low noise levels, $D_{0}\ll \Delta V$ , particle diffusion on a periodic substrate is reduced. Indeed, in the absence of SR, the MFPT for a low‐noise particle to jump out of a local minimum, say, for the process $x_{0}\to x_{0}\pm x_{L}$ , is represented by the Kramers’ time,^[9]^ $T_{K}=\left(2\pi /\omega_{0}^{2}\right)\exp\left(\Delta V/D_{0}\right)$ . For a particle under resetting the jumping mechanism requires that $\left\langle \tau \right\rangle >T_{K}$ , that is a sufficiently large $\left\langle \tau \right\rangle$ . Since in both panels of Figure 3 *τ*_0_ was taken quite small, $\omega_{0}^{2}\tau_{0}<1$ , the only way to obtain an appreciable drift at low noise was to lower *β* close to 1, see Eq. (8). Rectification appears to be optimal when $\left\langle \tau \right\rangle$ grows comparable with *T_K_*. On the contrary, for large *β*, $\left\langle \tau \right\rangle \to \tau_{0}$ and is, therefore, too small; vice versa, for β<1 , as mentioned above, $\left\langle \tau \right\rangle$ diverges and large SR times tend to suppress ratcheting.^[15]^ This argument explains the resonant profile of the low‐noise curves *v* versus *β* in Figure 3 and the shift of their maxima toward higher *β* values with increasing *D*_0_.

The positive, though small values of *v* for β<1 in Figure 3 indicate that rectification occurs also for $\left\langle \tau \right\rangle =\infty$ . To clarify this issue in the inset of Figure 3(b) we plotted the reciprocal of *v* computed as the average time, *T_m_*, the particle takes to jump *m* wells to the right divided by the distance, *mx_L_*, between them. We observe immediately that this ratio diverges as *β* approaches 1/2 from above. To support this numerical finding we also computed the distribution of the FPT's for the process $x_{0}\to x_{0}+mx_{L}$ (not shown). For β<1/2 we recovered power‐law distributions similar to those displayed in Figure 2(a) in the absence of substrate potential, $V\left(x\right)\equiv 0$ . This led us to conclude that *T_m_* diverges and *v* vanishes for β→1/2+ .

## 5 Diffusion Under Stochastic Resetting

We consider now the particle's diffusion. In the absence of a substrate potential, $V\left(x\right)\equiv 0$ , SR confines the particle around its injection point, *x*_0_, as apparent from Eqs. (6) and (9) and, therefore, its effective diffusion constant, *D*, defined in Eq. (4), is identically zero. On the contrary, in the presence of a rectifying substrate, the particle's drift is characterized by the net speed, *v*, investigated in Sec. 4, and its MSD, $\left\langle \Delta x^{2}\left(t\right)\right\rangle$ , defined in Eq. (4). For exponential and periodic SR time distributions, we showed in Ref. [13] that diffusion is normal for all values of $\left\langle \tau \right\rangle$ , that is $\left\langle \Delta x^{2}\left(t\right)\right\rangle =2Dt$ . We also estimated the fitting parameter *D* as a function of $\left\langle \tau \right\rangle$ . In particular, for $D_{0}\gg \Delta V$ , SR affects the particle's diffusivity to a small extent, $D≃D_{0}$ , whereas, in the opposite limit, $D_{0}\ll \Delta V$ , $D=x_{L}^{2}/2T_{K}\left(\left\langle \tau \right\rangle \right)$ , with $T_{K}\left(\left\langle \tau \right\rangle \right)$ denoting the Kramers’ time for the exit process $x_{0}\to x_{0}\pm x_{L}$ under resetting with average SR time $\left\langle \tau \right\rangle$ . The limit $T_{K}\left(\left\langle \tau \right\rangle \to \infty \right)$ coincides with the standard Kramers’ time, *T_K_*, reported above, while for estimates of $T_{K}\left(\left\langle \tau \right\rangle \right)$ at small $\left\langle \tau \right\rangle$ the reader is referred to Ref. [13].

Our new numerical results for the MSD of a particle diffusing under resetting with the Pareto time statistics of Eq. (3) are summarized in Figure 4. For the entire range of the tail index, *β*, the particle's MSD follows the normal diffusion law of Eq. (4), with numerical fitting parameter *D* reported in panel (b). For large *β*, $\left\langle \tau \right\rangle ≃\tau_{0}$ , so that, for $D_{0}\ll \Delta V$ , one recovers the well‐known result, $D≃x_{L}^{2}/2T_{K}\left(\tau_{0}\right)$ , with $T_{K}\left(\tau_{0}\right)$ denoting the Kramers’ time in the presence of SR with time constant *τ*_0_.^[13]^ In the opposite regime of $D_{0}\gg \Delta V$ , the potential barriers exert a negligible confining action, so that one recovers the vanishing substrate limit with $D≃D_{0}$ .^[13]^ Vice versa, for β<1/2 we already know that SR has no bearing on the particle's dynamics because $\left\langle \tau \right\rangle =\infty$ , so that we expect that $D≃x_{L}^{2}/2T_{K}$ , with *T_K_* denoting the Kramers’ time in the absence of SR (i. e., *D* grows independent of *τ*_0_). We recall that for large values of *β* and *D*_0_, namely for $\left\langle \tau \right\rangle$ ≥*T_K_*, $T_{K}\left(\tau_{0}\right)$ can be approximated to *T_K_*, which explains why in such conditions *D* is seemingly constant over the entire *β* range [see curves in Fig. 4(b) for *D*_0_ *=*1]. Of course, also for β≫1 , decreasing *τ*_0_ makes the Kramers’ time under resetting, $T_{K}\left(\tau_{0}\right)$ , grow exponentially large and, consequently, *D* is suppressed.

Most notably, the MSD deviates for the normal diffusion law of Eq. (4) for β→1+ , namely as $\left\langle \tau \right\rangle$ of Eq. (8) starts diverging. We remind that this is the *β* range where rectification is optimal (Figure 3), thus providing another instance of the connection between drift surge and excess diffusion.^[21]^ In this case the correct diffusion law reads $\left\langle \Delta x^{2}\left(t\right)\right\rangle \propto t^{\alpha }$ , with *α* as large as 1.76 for β=1.1 . Indeed, the *D* values reported in Figure 4(b) for 0.7<β<1.4 were fitted over the linear branches of the curves $\left\langle \Delta x^{2}\left(t\right)\right\rangle$ versus *t* (i. e., for t<50 ), for the sake of a comparison.

This result is unusual and has no counterpart in the current Brownian ratchet literature.[^15^, ^16^, ^17^, ^18^] To support this numerical finding we ran several checks. We preliminary noticed that (i) increasing the distribution scale parameter, *τ*_0_, namely for larger $\left\langle \tau \right\rangle$ values, the diffusion exponent decreases; (ii) the occurrence of superdiffusion is largely independent of the noise level. These remarks confirm that superdiffusion is related to rectification. Then, we truncated the Pareto distribution of Eq. (3) at $\tau =\tau_{\max}$ , meaning that the power‐law decay of $\rho \left(\tau \right)$ was restricted to the range [$\tau_{0},\tau_{\max}]$ . The effect of this change is displayed in the inset of Figure 4(a). For β=1.1 and sufficiently large values of $\tau_{\max}$ , superdiffusion still sets on, but only for $t<\tau_{\max}$ ; for $t>\tau_{\max}$ one recovers normal diffusion with α=1 . This is a clear indication that superdiffusion is related to the fat tails of the SR time distribution: cutting the $\rho \left(\tau \right)$ tails off suppresses superdiffusion.

**Figure 5:**
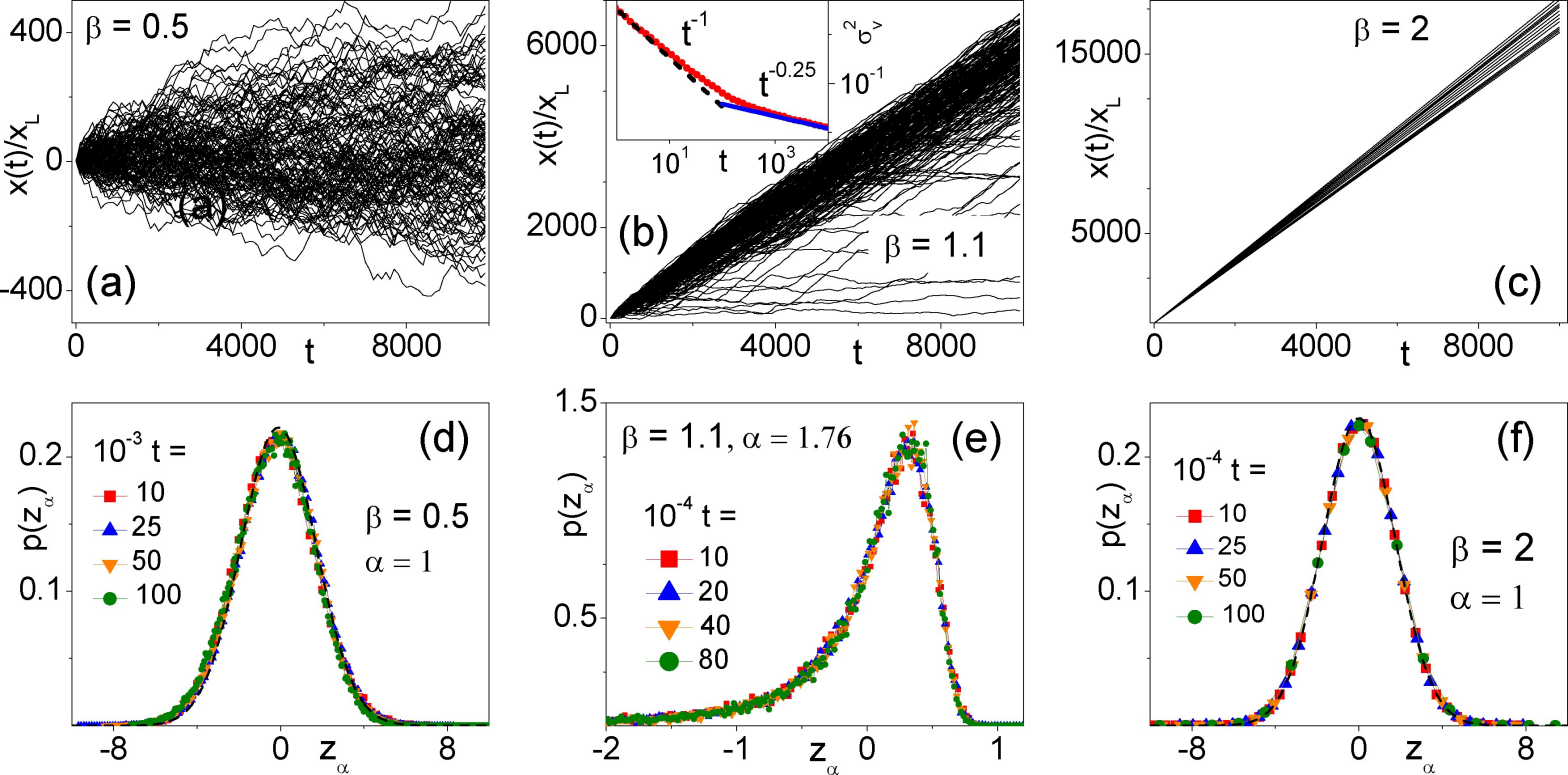
Trajectory statistics for a particle diffusing in the potential *V* (*x*) with *x_L_* =1 and Pareto *τ* statistics: (a)‐(c) trajectory samples for *D*_0_=2, *τ*_0_=0.01 and *β*=0.5 (no rectification, normal diffusion), 1.1 (optimal rectification, superdiffusion), and 2 (low rectification, normal diffusion). The inset in (b) is the variance of the time dependent trajectory drift speeds, $\sigma_{\upsilon }^{2}\left(t\right)$ (see text); (d)‐(e) corresponding pdf's of the particle positions at time, *t*, reported in the legends. The variable *x* has been rescaled to *z*_*α*_=(*x − (x)*)*/t* ^*α/*2^. The Gaussian (dashed) curves (4*πD*)^*−*1*/*2^ exp($-z_{1}^{2}$ */*4*D*) in (d) and (f) are reported for a comparison. The values of *α* and *D* used here have been extracted from the MSD data in Figure 4.

Another check is illustrated in Figure 5, where in panels (a)‐(c) we plotted trajectory samples of a particle under resetting respectively with β=0.5 (no drift), β=1.1 (optimal drift), and β=2 (small $\rho \left(\tau \right)$ tail effect). At different times, *t*, we computed the corresponding asymptotic distributions of the particle's position, *x*. In panels (d)‐(f) the variable *x* was rescaled as $x\to z_{\alpha }=\left(x-\left\langle x\right\rangle \right)/t^{\alpha /2}$ , with *α* the diffusion exponent introduced above. For a given *β*, all distributions collapse on a *unique* curve. When the diffusion is normal, α=1 , namely for β=0.5 and 2, such a curve is the Gaussian, $p\left(z_{1}\right)={\left(4\pi D\right)}^{-1/2}\exp\left(-z_{1}^{2}/4D\right)$ , with the appropriate diffusion constant *D* reported in Figure 4(b). In the presence of superdiffusion, i. e., for β=1.1 , the distribution $p\left(z_{\alpha }\right)$ of Figure 5(e) is strongly skewed with a long negative tail associated with the trajectories of Figure 5(b) that appear to diffuse very slowly, if at all. As there α=1.76 , the corresponding *x* distributions clearly broaden faster than linearly with time, and this mechanism is responsible for the detected superdiffusive effect. To support this observation more quantitatively, we computed the drift speeds of the trajectories sampled in Figure 5(b) as functions of time, $v_{i}\left(t\right)=x_{i}\left(t\right)/t$ , and determined their time‐dependent variance, $\sigma_{v}^{2}\left(t\right)=\left\langle v_{i}^{2}\left(t\right)\right\rangle -{\left\langle v_{i}\left(t\right)\right\rangle }^{2}$ , with $\left\langle ...\right\rangle$ denoting stochastic averages taken over an ensemble of $N=3\times 10^{3}$ trajectories. As illustrated in the inset of Figure 5(b), $\sigma_{v}^{2}\left(t\right)$ decays with time according to two distinct power laws, *t* ^−1^ and $t^{\alpha -2}$ , respectively at shorter and larger times. This is consistent with the relevant diffusion exponents of Figure 4(a), because, by the same argument, the corresponding MSD would amount to $\left\langle \Delta x^{2}\left(t\right)\right\rangle =\sigma_{v}^{2}\left(t\right)t^{2}$ .

## 6 Conclusions

In conclusion we have shown that randomly motile particles can propel themselves on a spatially asymmetric substrate by autonomously regulating their own internal motility for maximum efficiency. For fat‐tailed distribution of the activation times, the particle drift can turn superdiffusive. The interest of this conclusion goes beyond the context of SR, as discussed below.

The SR protocol of Sec. 2 can be readily generalized to the more realistic case when a finite latency time, *τ_l_*, is required before the reset particle is allowed to restart. As discussed in Ref. [13], this basically amounts to replacing $\left\langle \tau \right\rangle$ with $\left\langle \tau \right\rangle +\tau_{l}$ , thus leaving the overall SR properties unchanged. However, this variation of our model lends itself to an alternative physical interpretation. Consider the LE (1) with the potential of Eq. (2) but, instead of implementing the SR protocol with latency time *τ_l_*, we now assume a dichotomic noise strength, $D_{0}\left(t\right)$ , whereby *D*_0_ *=*0 for a fixed time interval, *τ_l_*, and $D_{0}\left(t\right)=D_{0}$ for a random time interval, *τ*, with Pareto distribution $\rho \left(\tau \right)$ . The resulting LE describes a special case of flashing ratchet^[16]^ with random cycle $\tau_{l}+\tau$ . This means that, as a side result, we have proven that under certain conditions, flashing ratchets with fat‐tailed time distributions may exhibit superdiffusive dynamics, an occurrence never reported in the earlier literature on autonomous particle transport.[^15^, ^22^]

## Conflict of Interests

The authors have no conflicts to disclose.

7

## Data Availability

The data that support the findings of this study are available within the article.
